# Clinical Presentation and Incidence of Anaerobic Bacteria in Surgically Treated Biliary Tract Infections and Cholecystitis

**DOI:** 10.3390/antibiotics10010071

**Published:** 2021-01-13

**Authors:** Jens Strohäker, Lisa Wiegand, Christian Beltzer, Alfred Königsrainer, Ruth Ladurner, Anke Meier

**Affiliations:** 1 Department of General, Visceral and Transplantation Surgery, University Hospital of Tuebingen, 72076 Tuebingen, Germany; Lisa.Wiegand@med.uni-tuebingen.de (L.W.); ChristianBeltzer@bundeswehr.org (C.B.); Alfred.Koenigsrainer@med.uni-tuebingen.de (A.K.); Ruth.Ladurner@med.uni-tuebingen.de (R.L.); Anke.Meier@med.uni-tuebingen.de (A.M.); 2 Department of General, Visceral and Thoracic Surgery, German Armed Forces Hospital of Ulm, 89081 Ulm, Germany

**Keywords:** anaerobic infection, cholecystitis, cholangitis, biliary tract infection

## Abstract

(1) Background: Cholecystitis and cholangitis are among the most common diseases treated by general surgery. Gallstones lead to inflammation and bacterial infection of the biliary tract. Biliary infections can lead to live threatening bacteremia and liver abscesses. The true role of anaerobes remains unclear. (2) Methods: We retrospectively analyzed bacterial cultures from biliary samples obtained from bile ducts and gallbladders at our tertiary care center. Patient characteristics and clinical outcomes were analyzed. (3) Results: In our database of 1719 patients, 365 patients had microbial testing, of which 42 grew anaerobic bacteria. Anaerobes were more frequently cultured in patients with hepatic abscesses and gallbladder perforation. These patients were older and had more comorbidities than the control group. The overall outcomes of all patients were favorable and the resistance rate to commonly used antibiotics remained low. (4) Conclusions: Anaerobes in biliary tract infections appear to be underdiagnosed and more prevalent in the elderly with advanced disease. Due to low antibiotic resistance, the combination of source control and adjunct anti-infective treatment leads to favorable outcomes.

## 1. Introduction

Cholecystitis and cholangitis are amongst the most common reasons for admission to general surgery departments [1]. Frequently, they are caused by gallstones irritating the gallbladder or bile duct epithelium which can lead to biliary obstruction, acute inflammation, and secondary bacterial or fungal infection. While acalculous cholecystitis exists, it is far less common than calculous cholecystitis and is frequently treated non-surgically [2].

When patients present with symptoms of acute cholecystitis, guidelines recommend performing an urgent cholecystectomy (CCE) within 72 h of symptom onset [3]. Patients usually receive at least one dose of antibiotic as a perioperative single shot. For mild and moderate acute cholecystitis, there is no evidence supporting prolonged antibiotic treatment [4]. Thus, the Tokyo Guidelines of 2018 (TG2018) recommend antibiotic treatment to be discontinued 24 h after source control has been achieved [5]. If there is severe cholecystitis or complicating factors such as perforation, peritonitis, or ongoing septicemia, the duration of antibiotic treatment may be prolonged up to the seventh postoperative day [5]. In cholangitis, antibiotic treatment should be withheld once the focus has been resolved. It may be continued if the anatomical obstruction of the bile duct cannot be cleared immediately. The pathogens implicated range widely; they can be upper or lower gastrointestinal bacteria which may be Gram positive, Gram negative, anaerobic, or aerobic. Most commonly enteric bacteria are isolated from biliary specimens [6]. While this list of bacteria is led by *Escherichia coli*, Enterococcus species and oral Streptococcus species, the reported rate of anaerobes varies between 0 and 23% [5,7,8]. To date, the gold standard of microbiological diagnostics is the culturing of the pathogens. Bile itself is considered toxic to most bacteria, therefore bile cultures frequently do not yield proper results [9]. Therefore, newer diagnostic tools are being developed. Alternative testing modalities (such as eubacterial polymerase chain reaction (PCR) or next generation sequencing (NGS)) are emerging, and are striving to replace bacterial cultures as the gold standard [8]. In the era of fast-track surgery, patients are frequently discharged before complete microbiological results are present. This is especially true for the slowly growing anaerobic strains. Knowing which organisms to cover empirically reduces unnecessary antibiotic use, thus potentially preventing side effects and limiting costs.

Uncomplicated cholangitis or cholecystitis were frequently treated with fluorquinolones and metronidazole due to their widespread availability, low rates of allergies, and potential to be administered both intravenously and orally. After the worldwide recommendation [10,11] to reserve fluorquinolone therapy for severely ill patients due to infrequent but severe side effects, the focus of anti-infective therapy has shifted back to aminopenicillins and cephalosporins.

Antibiotic treatment regimens vary across the globe, due to the local availability of certain antibiotics and local resistance rates. The 2018 Tokyo guidelines recommend the choice and duration of antibiotics based on the severity of disease and intraoperative findings [5]. Given the broad spectrum of potential bacterial culprits, the choice of antibiotics also needs to be broad, with both gram positive and gram negative coverage for aerobe and anaerobe organisms. In order to reduce polypharmacy, ampicillin/sulbactam or amoxicillin/clavulanic acid are the drugs of choice.

A review by Thabit et al. summarized the current knowledge on the bioavailability of nearly every commonly used antibiotic in the gallbladder, bile duct, and bile itself. They reviewed several studies that published unpromising results for ampicillin’s and amoxicillin’s biliary concentrations, and thus their potential to treat biliary anaerobic infections is questionable [12].

Therefore, we need better diagnostic tools to guide empiric antibiotic selection for the treatment of cholecystitis and cholangitis.

The goal of this study was to evaluate the incidence and clinical role of anaerobic bacteria in patients suffering from cholecystitis and cholangitis.

## 2. Results

### 2.1. Clinical Characteristics

We analyzed the data of all consecutive cholecystectomies from our clinical database, which were performed as independent procedures at the Department of General, Visceral and Transplantation Surgery of the Tuebingen University Hospital, Germany, from 2014 to early 2020. Overall, a total of 1719 cases of cholecystectomies were identified from the database. In uncomplicated cases, cholecystectomy was performed minimally invasive and no specimens were collected. These patients received a single dose of perioperative cephalosporin and were usually discharged on two days postoperatively after an uneventful course. If there was concern for more severe disease, the surgical team was encouraged to send bile for culturing to potentially guide anti-infective treatment. In the present study, all patients who had specimens sent for microbiological testing were identified. There were 365 patients that met the inclusion criteria. Approximately half of these patients were male (50.7%) and half were female (49.3%), with a median age of 66 years (range 20 to 97)—for more details see Table 1. Microbiological specimens were either collected endoscopically during preoperative endoscopic retrograde cholangiopancreaticography (ERCP), during the surgical procedure by puncturing the gallbladder with a sterile cannula, or with a swab from the gallbladder epithelium after resection of the gallbladder.

Antibiotic treatment was selected based on the diagnosis. Patients that presented with cholangitis were frequently treated with antibiotics leading up to the cholecystectomy, followed by few postoperative doses, whereas patients with acute cholecystitis frequently had urgent surgery followed by a longer course of postoperative antibiotics.

There were 42 patients whose bile specimens grew anaerobic bacteria during their clinical stay. The patient group that had positive anaerobic cultures and those who did not were similar with respect to gender distribution, body mass index (BMI) and age, although patients with anaerobic infections were older than those who did not (72 vs. 66 years, *p* 0.095). Operating times were similar (96 vs. 98 min) as well as the operative strategy (74 vs. 77% were performed laparoscopically). We hypothesize that the age discrepancy may be the reason for the increased percentage of hypercholesterinemia in the anaerobic group. There was a significant difference in the presence of gallbladder perforation and pericholecystic abscesses in the group that had a culture-positive anaerobic infection (43 vs. 27%, *p* 0.028). The rate of patients that had undergone preoperative ERCP was considerably higher in the group that had positive anaerobic cultures (45 vs. 19%, *p* 0.000). Procedure related complications (e.g., wound infections, bile leaks and the need for additional interventions) were similar in both groups (17 vs. 11%). For details, see Table 2.

**Figure 1:**
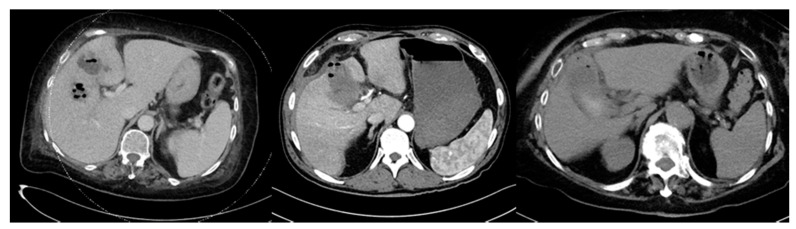
Shows three individual patient’s CT scans with gas bubbles inside the gallbladder as well as one with gas bubbles inside an adjacent liver abscess (far left).

Abdominal ultrasound is the gold standard imaging modality for diagnosing acute cholecystitis. However, in cases of septicemia or severely ill patients, a computer tomography (CT) can be helpful to guide operative treatment approaches. Of our 42 patients, 28 had a CT scan prior to gallbladder removal. We screened these for CT signs of anaerobic infection (e.g., gas bubbles within the gallbladder or adjacent abscesses). Three of the patients showed intracholecystic air, raising concerns for anaerobic infection (Figure 1). There was no specific bacterial strain associated with gas bubbles on the CT scan.

### 2.2. Microbial Growth

Of the 365 patients that had intraoperative microbiological testing, 174 (49.6%) showed bacterial growth, whereas 178 of the cultures remained sterile and thus yielded no results. The most common site of bacterial origin was the gastrointestinal (GI) tract with 123 (70.7%) patients with at least one strain of enteric bacteria. Twenty-eight cultures yielded solely oral bacteria (16.1%). Fourteen specimens grew only skin bacteria (8.0%), leaving concern for contamination. Nine patients had a mixed growth of enteric and skin bacteria (5.2%). While *E. coli* (*n* = 59) and Klebsiella spec. (*n* = 24) were the most common Gram negative isolates, they were almost equaled in numbers by *E. faecalis* (*n* = 30) and *E. faecium* (*n* = 28).

In this study, 47 non-repetitive anaerobic microorganisms were cultured from samples of 42 patients. Pure anaerobic cultures with only one strain were rare, and only occurred in 7 patients (17%). The 35 remaining patients had polymicrobial cultures with one or more aerobic or anaerobic bacteria. Five patients showed at least two different anaerobic strains (12%). The most common isolates were Bacteroides fragilis (*n* = 8, 17%), Clostridium perfringens (*n* = 7, 14.9%), and Actinomyces odontolyticus (*n* = 7, 14.9%).

Fourteen out of these 42 patients (33%) had positive cultures from a preoperative ERCP procedure due to cholangitis/choledocholithiasis. Here, the most common isolate was Actinomyces odontolyticus (*n* = 6) followed by Cutibacterium acnes (*n* = 2) (Table 3).

According to protocol, antibiotic resistance testing in anaerobes is only performed at our hospital once there are pure monocultures and/or clinically highly significant isolates (e.g., blood cultures, brain abscesses) identified. In all other cases, a general recommendation is given to the clinician about which antibiotics are recommended for which bacterial strains based on general mechanisms of resistance as well as locally observed resistance rates. We therefore collected an overview of all isolates that were tested from 2013 to 2019 (2020 was not yet available). For details, see Table 4.

Actinomyces species were tested for amoxicillin/clavulanic acid and meropenem. Cutibacterium acnes strains were tested for penicillin G, clindamycin, amoxicillin/clavulanic acid, meropenem and metronidazole. All other available strains were tested for penicillin G, clindamycin, amoxicillin/clavulanic acid, meropenem and metronidazole. According to the aforementioned empirical treatment recommendations, amoxicillin/clavulanic acid or piperacillin/tazobactam should have treated 42 of 47 strains (89.4%). Metronidazole treated 35 of 47 strains (74.5%).

### 2.3. Antibiotic Treatment

Thirty-five of the 42 patients were treated with more than a perioperative single shot antibiotic (83%). Median antibiotic treatment lasted for five days (standard deviation (SD) 2.0, range 1–11 days). The most common primary regimens were ciprofloxacin + metronidazole (43%); followed by piperacillin/tazobactam (17%); meropenem (10%); 3rd generation cephalosporin/metronidazole (7%); and ampicillin/sulbactam (7%).

Septicemia was seen in eleven (26%) patients in this study. According to the 2018 Tokyo guidelines, eight patients suffered from severe cholecystitis and five from severe cholangitis (two patients overlapping and suffering from both).

There were five infectious complications, all of which were superficial wound infections. Four out of five were found in the patients that had undergone laparotomy. The remaining patient suffered from a port-site infection after laparoscopic cholecystectomy. One patient underwent conventional cholecystectomy for necrotizing cholecystitis. She received five days of ciprofloxacin post-operatively, but no antibiotic covering anaerobic bacteria. Her intraoperative swabs showed growth of *B. intestinalis*, *B. vulgatus* and *B. uniformis*. On postoperative day seven (the day of discharge), she developed a small wound abscess which was drained. From that abscess, four different anaerobic strains including large amounts of B. thetaiotaomicron and Peptoniphilus gorbachii were grown. Whether the addition of metronidazole or a different antibiotic agent could have prevented abscess formation remains unclear.

This does not only represent biliary tract infections.

## 3. Discussion

Symptomatic cholecystolithiasis, choledocholithiasis and acute cholecystitis are some of the most common surgically treated diseases in general surgery departments worldwide. In most cases, cholecystitis is caused by gallstones with subsequent bacterial infection of the gallbladder and may be accompanied by biliary tract infection. Both can lead to septicemia and death. Historically empiric antibiotic coverage included substances that specifically treated aerobic and anaerobic bacteria [13].

The role of anaerobes in cholecystitis has been discussed repeatedly. There are sufficient data to support the fact that there is no benefit from administering prolonged perioperative antibiotics in mild cholecystitis [14]. Furthermore, the recommendation to cover anaerobic bacteria in mild and moderate cholecystitis is not upheld by the 2018 Tokyo guidelines. However, they provide a somewhat conflicting recommendation when it comes to anaerobic treatment. For moderate or severe cholecystitis, they recommend to either use piperacillin/tazobactam, a carbapenem, or a third/fourth generation cephalosporin without the addition of metronidazole. Metronidazole is only recommended when bilioenteric anastomosis is present. This does only apply to cholangitis because the gallbladder is always removed simultaneously when patients undergo bilioenteric anastomosis (e.g., in liver transplantation or, duodeno-cephalo-pancreatectomy) [5,15].

Due to preoperative antibiotic therapy and bile toxicity to bacteria, bile cultures are frequently negative, and their yield of anaerobic strain data is even lower. Of the 365 patients from whom microbiological samples were collected in our institution, we were only able to culture bacteria in approximately 50%, which is slightly more (numerically) than 10% of the overall cohort of 1719 patients. This is similar to other previously described cohorts [16,17]. In our dataset, about 10% of patients with positive cultures showed anaerobic growth, and these positive anaerobic cultures were frequently polymicrobial, including both aerobic and multiple anaerobic strains. Monoculture was exceedingly rare. A study from Norway performed next-generation sequencing on bile aspirates and showed up to 23% of anaerobic bacteria from these samples [8]. Sterile puncturing of the gallbladder appears to be the most reliable mode of sampling specimen for anaerobic cultures (and likely aerobic cultures too). Unfortunately, we are unable to report numbers of suboptimal specimen sampling and/or handling prior to microbiological workup, which may be a potential bias.

Fast-track surgery depends on targeted empiric treatment accompanied by early knowledge of microbiological culprits. Therefore, it has to be discussed whether anaerobic culture is the optimal tool to guide empiric anti-infective treatment once patients are at risk for anaerobic infections.

The TG2018 leave it in physicians’ hands whether to prolong anti-infective treatment in mild or moderate cholecystitis when perforation, emphysematous or necrotizing cholecystitis are detected. Given the higher rate of concomitant anaerobic infections and perforations/abscesses, we suggest covering anaerobes in these patients until final microbiological results are available.

To our knowledge, this is the first study to specifically describe a series of perioperative anaerobic infections in patients undergoing cholecystectomy for symptomatic cholelithiasis or cholecystitis. Even though anaerobes were technically present in the more complicated cases (abscesses, perforations), the outcome of the patients was favorable; in our cohort, all 42 patients were successfully treated with perioperative antibiotic therapy combined with endoscopic and operative resolution of the infectious focus. The percentage of patients that had undergone ERCP prior to CCE was higher in the anaerobic group (45 vs. 19%). Preoperative biliary occlusion due to choledocholithiasis may be a risk factor for the development of anaerobic infection and should set at a low threshold for perioperative antibiotic coverage of anaerobes.

There appears to be a different spectrum of bacteria in cholangitis compared to cholecystitis, with a higher prevalence of Gram positive oro-pharyngeal strains in cholangitis. Whether these bacteria play a major role in the pathogenesis of cholangitis or are contaminants remains uncertain. Given that sampling of bile during endoscopy cannot be perfectly sterile, there may be concern for contamination.

We isolated mostly Bacteroides strains and Clostridium perfringens from gallbladder samples, whereas oral Cutibacterium and Actinomyces species were more common in ERCP-samples. The isolated pathogens are different from what has been published by other groups. Bacteroides were the most common pathogens in our cohort, however Dyrhovden et al. did not report a single case of Bacteroides, either in biliary culture or from biliary sequencing [8]. The same study reported a high prevalence of Fusobacterium DNA in their samples, however failed to culture them. To our knowledge, our study is the first to present a case of necrotizing cholecystitis caused by culture-proven Fusobacterium nucleatum.

We presented one case of a patient with necrotizing cholecystitis who had three different strains of anaerobic bacteria isolated and whose case was correctly classified as moderate cholecystitis according to TG2018. She did not have empiric anaerobic coverage postoperatively and was also not started on an antibiotic for anaerobes when the final culture report was released five days postoperatively, when the infection was considered resolved. On postoperative day seven, the patient developed a wound infection caused by anaerobes. Whether the infection could have been prevented is unclear. We believe that the least the surgeon should do is to inform the patient about such results in order to educate them on what to look out for in the postoperative period.

In the light of the amount of time anaerobic cultures still take to complete, next generation sequencing—once it is made widely available—could offer empiric antibiotic guidance within hours of surgery and thus could avoid both unnecessary doses and omission. It is crucial to know one’s local resistance rates to antibiotics not only for aerobic bacteria, but also for anaerobic bacteria, because while we are still in a position of low rates of anaerobic antibiotic resistance, the number appears to be increasing in enteric bacteria annually [18,19]. Unfortunately, more recent data reporting on resistance rates in anaerobic bacteria are scarce [20,21].

Given its excellent penetration into bile and the susceptibility of both Gram positive and Gram negative bacteria as well as almost all anaerobes, piperacillin/tazobactam is an excellent choice in moderate or severe cholecystitis [22,23]. Alternatives exist according to the 2018 Tokyo guidelines [5]. Ciprofloxacin and metronidazole were formerly the widespread standard for oral antibiotic treatment in biliary infections. With the recommendation to avoid fluorquinolones [11], we are revisiting beta-lactams to treat abdominal infections. Unfortunately, there are conflicting data on the efficacy of aminopenicillins and oral cephalosporins. Although studies support the use of cefuroxime (with metronidazole) given their excellent excretion into the biliary tract [24], we frequently cultured enterococci and unsusceptible Gram negative rods in our cultures, which is supported by other studies [8]. Therefore, cefuroxim can only be recommended in mild cases and based on local susceptibility rates which can be discussed with the local antibiotic stewardship team.

While amoxicillin/clavulanic acid has a similar activity towards anaerobes, its role as an oral step-down alternative to piperacillin/tazobactam is debatable due to its supposed lack of biliary concentration in the gallbladder and bile duct. Amoxicillin/clavulanic acid has been shown to be inferior to ceftibuten and non-superior to a placebo in cholecystitis treatment [12,25].

### Strengths and Limitations

We presented the first study to focus solely on the incidence and management of anaerobic infections in a large cohort of patients that underwent laparoscopic cholecystectomy at a single institution. Over 1700 patient charts were reviewed for microbiological testing and we were able to demonstrate anaerobic bacterial growth in 42 out of 365 cultures. Anaerobic bacteria appeared to be more common in patients that had undergone preoperative ERCP. Patients referred to our department nearly always undergo CCE; therefore, we are unable to present data on the outcome of patients that were treated purely medically without surgical intervention or drainage.

Due to the retrospective aspects of this study, we are unable to correlate our culture results with that of next generation sequencing. Given that we isolated a distinctly different bacterial spectrum than was presented by Dyrhovden et al. in their NGS study, we are convinced that larger prospective, multicentric trials with highly standardized sampling techniques are needed to investigate the true impact of anaerobic infections of the biliary tract. In our cohort, the patients suffering from anaerobic infections appeared to be older and appeared to present with perforations more frequently. However, larger trials are needed to identify risk factors for anaerobic infections in order to optimize empiric antibiotic treatment in biliary tract infections.

## 4. Materials and Methods

### 4.1. Data Acquisition

We retrospectively screened our hospital information system for all patients who underwent CCE at the department of General, Visceral and Transplantation Surgery of the University Hospital of Tuebingen, Germany. All adult patients (age ≥ 18 years) who underwent cholecystectomy as an individual procedure at our center between January 2014 and June 2020 were included in the final analysis. Patients that had CCE as part of another procedure (i.e., liver resection or pancreatic surgery) were excluded. The medical reports of these patients were screened for perioperative biliary cultures. Bile cultures were sent at the surgeon’s discretion, most commonly when there were clinical signs of cholangitis, cholecystitis or intraoperative findings of abscesses or perforation. The study was performed on a consecutive database and was approved by the local ethics committee under the reference numbers 502/2019/BO2 and 715/2020BO2.

### 4.2. Clinical Definitions

Acute cholecystitis and cholangitis were classified according to the 2018 Tokyo Guidelines. Acute cholecystitis is defined as the inflammation of the gallbladder with typical symptoms of right upper quadrant pain, elevated inflammatory parameters and ultrasonographic signs of gallbladder inflammation [26].

### 4.3. Isolation and Identification of Strains

All samples were sent for both aerobic and anaerobic culture. For the anaerobic cultures, we used a custom-built highly enriched and supplemented sheep-blood agar. The exact method has been described by our microbiology department [27]. The isolates were identified using MALDI-TOF mass spectrometry. In one patient, a strain of Clostridium perfringens was identified using sequencing or a eubacterial PCR.

### 4.4. Statistics

Comparison between groups was carried out by Chi-squared test or Fisher’s exact test for nominal variables, and Mann–Whitney U test or Kruskal–Wallis test for continuous variables, as appropriate. A probability of less than 0.05 was considered to be statistically significant. All *p*-values reported are results of two-sided testing. Where needed, Bonferroni-correction was applied. Statistical analysis was carried out using IBM SPSS Statistics for Windows, Version 26.0 (IBM Corp., Armonk, NY, USA).

## 5. Conclusions

This study is the first to report on a cohort of patients who underwent cholecystectomy and were diagnosed with anaerobic infection of their biliary during their hospital stay. Anaerobic bacteria appear to be neglected pathogens due to the limited capability to grow these bacteria and to acquire proper antibiograms. We strongly believe that there is a need to better understand the growing rates of antibiotic resistance in abdominal anaerobic infections in order to use the latest diagnostic methods in order to guide empiric treatment. In our cohort, patients presented anaerobic infections more frequently at older ages and with advanced diseases. Preoperative ERCP also appears to be accompanied by positive anaerobic cultures. However, given the favorable outcome of our patients, obligatory anaerobic antibiograms do not (yet) to appear warranted in this population.

Although the outcomes of anaerobic infection are favorable, we strongly believe that anaerobic infections are a neglected disease. Given the potential underdiagnosis due to the low detection rates, we may miss preventable complications. Furthermore, widespread antibiotic resistance testing will be warranted in the future in order not to miss increasing rates of resistance.

## Figures and Tables

**Table 1:** The characteristics of all patients that had specimens sent for microbiological culture. * Urgent cases were defined as all patients that had to undergo cholecystectomy within 72 h of symptom onset.

Overall Patient Characteristics (*n* = 365)		
Gender m/f	185/180	50.7%/49.3%
Median age in years	66	Range 20–97
BMI in kg/m^2^	27	Range 17–60
Diagnosis		
Acute Cholecystitis	245	31.5%
Chronic Cholecystitis	115	67.1%
Tumor	5	1.5%
Preoperative endoscopic retrograde cholangiopancreaticography (ERCP)	79	21.6%
Urgent cases *	212	66.3%
Median operating time in minutes	98	Range 28–302
Median length of stay in days	3	Range 1–51
Laparoscopic cholecystectomy (CCE)	280	76.7%
Conversion to Open CCE	31	8.5%
Primary Open CCE	54	14.8%

**Table 2:** A comparison of the patients with anaerobic infection (first column) and the patients without anaerobic growth (second column).

Anaerobic vs. Aerobic Patients			
	Anaerobes (*n* = 42)	No Anaerobes (*n* = 323)	*p*
Gender m (Percentage)	21 (50%)	164 (51%)	1.000
Median age in years	72	66	0.095
Body mass index (BMI) in kg/m^2^	27	27	0.788
Preoperative ERCP	19 (45%)	60 (19%)	0.000
Surgical Diagnosis			
Acute Cholecystitis	29	216	0.778
Non-acute Cholecystectomy	13	30	
Perforation or Abscess	18 (43%)	85 (27%)	0.028
Median operating time in minutes	96	98	0.178
Median length of stay in days	4.5	3	0.151
Laparoscopic CCE	32 (76%)	248 (77%)	1.000
Conversion to Open CCE	3 (7%)	28 (8%)	
Primary Open CCE	7 (17%)	47 (15%)	
Complications	7 (17%)	36 (11%)	0.296
Diabetes	9 (21%)	73 (23%)	1.000
Hypercholesterinemia	14 (33%)	62 (19%)	0.034

**Table 3:** The isolated anaerobic strains. The bacteria are sorted within their species from the most frequent to the least.

Microorganisms Isolated from Biliary Specimens		
	*n*	%
*Bacteroides fragilis*	8	17.0
*Bacteroides uniformis*	4	8.5
*Bacteroides thetaiotaomicron*	4	8.5
*Bacteroides ovatus*	1	2.1
*Bacteroides intestinalis*	1	2.1
*Bacteroides vulgatus*	1	2.1
*Clostridium perfringens*	7	14.9
*Clostridium hatewayi*	1	2.1
*Actinomyces odontolyticus*	7	14.9
*Actinomyces naeslundii*	1	2.1
*Cutibacterium acnes*	4	8.5
*Prevotella* spec.	3	6.3
*Bilophila wadsworthia*	1	2.1
*Fusobacterium nucleatus*	1	2.1
*Slackia exigua*	1	2.1
*Bifidobacterium breve*	1	2.1
*Alloscardovia omnicolens*	1	2.1
Total	47	100

**Table 4:** Anaerobic strains that were cultured by the local microbiology department since 2014 and their antibiotic susceptibility rates.

Local Susceptibility Rates by Microorganisms in %						
	Penicillin G	Amox./Clav.	Clindamycin	Meropenem	Metronidazole	*n*
*Actinomyces* spec.	n.t.	100	100	100	0	34
*Cutibacterium acnes*	95.8	100	98.6	98.6	0	72
*B. fragilis*	0	94.1	70,6	85.7	97.1	35
*B.* species	0	88.8	77.8	100	100	9
*C. perfringens*	83.3	100	66.7	100	100	12
*C.* species	71.9	100	66.7	100	100	33
*Prevotella* species	33.3	100	55.6	100	55.6	9
*Fusobacterium nucl.*	100	100	20	100	100	5

## Data Availability

The data presented in this study are available on request from the corresponding author. The data are not publicly available du the pseudonymized character of the data.
